# 肺叶切除及肺段切除治疗早期非小细胞疗效对比研究进展

**DOI:** 10.3779/j.issn.1009-3419.2019.08.08

**Published:** 2019-08-20

**Authors:** 亮 陈, 文涛 方

**Affiliations:** 200030 上海，上海交通大学附属胸科医院 Department of Thoracic Surgery, Shanghai Chest Hospital, Shanghai Jiao Tong University, Shanghai 200030, China

**Keywords:** 肺叶切除, 肺段切除, 肺肿瘤, Lobectomy, Segmentectomy, Lung neoplasms

## Abstract

肺癌是全球高发病率及高死亡率的恶性肿瘤。随着低剂量螺旋计算机断层扫描应用于肺癌的筛查与诊断，早期肺癌的检出率得以提升，使得肺癌通过外科手术治愈的可行性大大提高。解剖性肺叶切除长期以来一直是治疗早期非小细胞肺癌的标准术式，但意向性肺段切除能否安全有效被应用仍是个备受争议的问题。本文对此问题进行探讨。

## Abstract (en)

Lung cancer is a worldwide malignancy with high morbidity and mortality in the world. With the application of low-dose spiral computed tomography in the screening and diagnosis of lung cancer, more lung cancers are detected at an early stage and cured by surgical treatment. Anatomical lobectomy has long been the standard procedure for the treatment of early stage non-small cell lung cancer. However, whether intentional segmentectomy can be more used remains controversial. And this paper focuses on the question.

## 1 前言

肺癌占癌症总发病人数的11.6%，占癌症总死亡人数的18.4%，是发病率及死亡率最高的恶性肿瘤^[1]^。早期肺癌主要采取手术切除肿瘤，以达到肿瘤学根治，预后较好。目前美国国立综合癌症网络（National Comprehensive Cancer Network, NCCN）指南推荐治疗早期非小细胞肺癌（non-small cell lung cancer, NSCLC）的首选术式是肺叶切除。但随着低剂量螺旋计算机断层扫描（low-dose computed tomography, LDCT）在肺癌筛查中的普及，早期肺癌的检出率得以提升^[2, 3]^，肺段切除也更多地被应用于外科治疗中。除了适用于术前心肺功能差，存在实施肺叶切除高危因素或禁忌的患者，NCCN指南指出对以下情况实施肺段切除是安全可行的：当结节 < 2 cm时，需满足以下三个条件之一，即病理为原位腺癌（adenocarcinoma *in situ*, AIS）、磨玻璃成分（ground glass opacity, GGO） > 50%、影像学监测下肿瘤倍增时间 > 400 d。理论上段切相对于叶切的优势在于更能减少术后肺功能损失，减少围手术期死亡率及并发症发生率等；缺点在于切除范围不足及淋巴结清扫不足而增加术后复发率及肿瘤相关死亡率。但这几点需随机对照临床试验（randomized controlled trial, RCT）证实。早在1995年北美肺癌研究组（Lung Cancer Study Group, LCSG）报道了一项关于T1N0M0 NSCLC术式比较的RCT结果，表明亚肺叶切除术后肿瘤相关死亡率比肺叶切除高50%，局部复发率高于肺叶切除3倍^[4]^，同时两者术后肺功能损失相近。但该项研究结果的推广存在一些局限性：相当一部分亚肺叶切除病例为楔切，而段切与叶切预后差异相对较小；纳入的病例均依据X线诊断，导致病理分期与临床分期符合率较低；当时的病理类型尚未进一步细分^[5, 6]^。目前正在进行的RCT如JCOG0802及CALGB140503结果尚未完善，所以段切能否安全有效地被应用于早期NSCLC的外科治疗存在争议。本文对肺段切除及肺叶切除肿瘤学、围手术期及功能学预后比较展开讨论。

## 2 肿瘤学预后

欲以段切替代叶切作为标准术式，首先需证明两者的肿瘤学结果相似。近年来已有多项回顾性研究表明早期NSCLC段切及叶切术后生存及复发并无显著差异^[7-10]^。

肿瘤大小是早期NSCLC预后的影响因素之一。大多数文献报道2 cm以下肿瘤行段切及叶切具有相近的肿瘤学效果^[11-13]^。Okada等^[14]^研究结果显示2 cm以下NSCLC行段切及叶切术后5年无病生存率（disease-free survival, DFS）（92.2% *vs* 91.5%, *P*=0.431, 6）与5年总生存率（overall survival, OS）（95.3% *vs* 93.9%, *P*=0.445, 9）无显著的统计学差异；Tsutani等^[15]^研究表明2 cm以下结节段切及叶切术后3年无复发生存率（recurrence-free survival, RFS）（90.9% *vs* 92.9%）及3年OS（95.7% *vs* 93.2%）无统计学差异。但Dai等^[16]^的研究认为肿瘤无论是 < 1 cm还是介于1 cm-2 cm之间，段切的OS及肺癌特异性存活率（lung cancer-specific survival, LCSS）均较叶切差。

术中淋巴结的切除数量一定程度决定了病理分期的准确性^[17]^。有研究^[9, 18]^表明段切与叶切的淋巴结切除总数及组数之间无差异。Mattioli等^[19]^认为对于2 cm以下NSCLC，段切相对叶切在N1（*P*=0.43）及N2（*P*=0.68）淋巴结检测均无差异。Khullar等^[20]^研究表明对于2 cm以下NSCLC，当满足切缘阴性及保证淋巴结切除数量，段切与叶切术后OS差异不大（HR=1.20, 95%CI: 0.86-1.67, *P*=0.277）。

以GGO成分为主的肿瘤大多为腺癌早期病变，如非典型腺瘤样增生（atypical adenomatous hyperplasia, AAH）、原位腺癌（adenocarcinoma i*n situ*, AIS）、微浸润腺癌（minimally invasive adenocarcinoma, MIA），或是以腺泡为主、以乳头为主、伴少量附壁结构的浸润性腺癌^[21, 22]^。这类肿瘤有惰性，段切预后相对较好^[23, 24]^。而以实性成分为主的肿瘤，有研究^[25, 26]^表明两种术式的肿瘤学预后相似。对于纯实性肿瘤，段切预后尚存在争议。Koike等^[27]^研究认为2 cm以下实性结节段切与叶切术后5年OS（85.2% *vs* 84.2%）、10年OS（65.5% *vs* 63.1%）、5年DFS（80.0% *vs* 76.9%）、10年DFS（63.8% *vs* 58.0%）无显著差异（OS: *P*=0.767; DFS: *P*=0.635），段切并非OS与DFS的独立危险因素。相反，Hattori等^[28]^发现2 cm以下实性结节段切较叶切会降低患者的3年RFS（82.2% *vs* 90.6%, *P*=0.048, 8），增大局部复发风险（20.7% *vs* 8.2%, HR=2.924, 95%CI: 1.115-7.792, *P*=0.029, 2）。

不同病理类型或腺癌亚型，段切的肿瘤学预后尚不明确。Veluswamy等^[29]^研究表明2 cm以下浸润性腺癌，段切术后OS及LCSS近似于叶切；若病理为鳞癌，则段切术后OS及LCSS劣于叶切。Yano等^[30]^的多中心回顾性研究表明，对于实性成分最长径/肿瘤最长径（consolidation tumor ratio, CTR）≤0.25的腺癌，肺段切除是安全的术式；对于鳞癌、大细胞肺癌、CTR > 0.25的腺癌，如无禁忌，应选择肺叶切除。在浸润性腺癌不同病理亚型中，伏壁样腺癌预后相对较好，腺泡样、乳头状预后次之，微乳头状、实体及黏液腺癌预后最差^[31-35]^。Dembitzer等^[36]^发现2 cm以下各亚型的腺癌段切及叶切并不存在生存差异（*P*=0.770, 4）。Xiao等^[37]^的一项前瞻性研究表明除了伏壁样腺癌，其他亚型并不适合行肺段切除，因非伏壁样腺癌段间淋巴结转移率相对较高（OR=12.18, 95%CI: 3.59-41.34, *P* < 0.001）。

综上所述，对于2 cm以下早期肺癌行段切与叶切在肿瘤学预后上可能并不存在显著差异，但仍需研究实性成分及病理类型对段切预后的影响。

## 3 围手术期比较

肺段切除较肺叶切除更需面临解剖的复杂性及变异问题、术中病灶精确定位及肺段边界的鉴别，故技术层面段切比叶切难度更大、要求更高，因此需证明相比于叶切而言段切同样安全可行。对于叶切及段切围手术期死亡率及并发症发生率，目前已发表的多数研究表明两者并无显著差异^[38, 39]^。Hwang等研究^[40]^结果表明叶切组与段切组在手术时间（*P*=0.47）、住院天数（*P*=0.31）、术后并发症（*P*=0.1）及死亡率（*P*=0.36）无显著差异。Miyasaka等^[41]^认为段切术后并发症发生与术中出血量相关（HR=1.014, 95%CI: 1.001-1.028, *P*=0.036），同时左上叶固有段切除术后有更高的并发症发生率（HR=9.783, 95%CI: 1.834-52.178, *P*=0.008）。Ueda等^[42]^对叶切及段切术后房颤发生进行单因素分析，结果表明术后房颤发生与年龄（*P* < 0.01）、心肌缺血史（*P*=0.03）、第1秒用力呼气容积（forced expiratory volume in one second, FEV_1_%）（*P* < 0.01）及手术方式（*P*=0.01）相关；进一步多因素分析表明高龄（HR=1.059, 95%CI: 1.015-1.106, *P* < 0.01）、肺叶切除（HR=5.734, 95%CI: 1.350-24.361, *P*=0.02）及FEV_1_% < 70%（HR=2.182, 95%CI: 1.067-4.461, *P*=0.03）是术后发生发颤的独立危险因素。目前两项进行的随机对照临床研究，JCOG0802试验叶切及段切的围手术期对比结果显示瘘或漏气在段切组发生率高于叶切组（6.5% *vs* 3.8%, *P*=0.04），多因素分析表明肺部并发症（包括漏气及脓胸）的独立危险因素是复杂段切（OR=2.07, 95%CI: 1.11-3.88, *P*=0.023）及超过20年包的吸烟史（OR=2.61, 95%CI: 1.14-5.97, *P*=0.023），其中复杂段切涉及除背段、左上叶固有段及舌段切除之外的段切^[43]^；CALGB140503试验^[44]^的事后分析结论表明2 cm以下早期肺癌行意向性肺段切除与肺叶切除在围手术期死亡率及并发症发生率上无显著差异，可见意向性段切治疗早期NSCLC是一种安全的术式。

## 4 肺功能保留

相较于叶切而言段切的优势在于肺功能的保留。理论上段切切除更少的肺组织，多项研究同样认为段切术后肺功能保留优于叶切^[45]^，但考虑到术后余肺功能存在代偿，故目前两种术式对肺功能的影响尚不明确^[46]^。Keenan等^[47]^对比了术后1年叶切及段切组用力肺活量（forced vital capacity, FVC）、FEV_1_、最大通气量（maximum ventilatory volume, MVV）、肺一氧化碳弥散量（diffusing capacity of the lungs for carbon monoxide, DLCO）的变化，发现叶切组这四个指标均较术前明显下降，而段切患者仅DLCO有明显下降。Gu等^[48]^认为段切减少了FVC损失（*P*=0.048），而非FEV_1_（*P*=0.273）或DLCO（*P*=0.293），而在平均每肺段损失的肺功能上，段切约高于叶切的2倍。通过放射性核素定量显像及定量CT可以检测局部肺叶的肺功能代偿情况，从而直观地评定两种术式对于肺功能的影响^[49, 50]^。Nomori等^[51]^利用单光子发射计算机断层成像术（single-photon emission computed tomography/computed tomography, SPECT/CT）测定全肺、病灶同侧非手术肺叶及对侧各肺叶的术前、术后6个月的FEV_1_值，发现两种术式术后对侧各肺叶的FEV_1_均有显著的增加（*P* < 0.001），而同侧非手术肺叶FEV_1_仅段切组有显著的增加（*P*=0.003），叶切组则没有显著改变（*P*=0.97）。相反，Suzuki等^[52]^的研究结果表明术后2个月内段切组的肺功能恢复显著优于叶切组（FVC: *P* < 0.001; FEV_1_: *P* < 0.01）；而术后6个月后，两组的肺功能改变无显著差异（FVC: *P*=0.96; FEV_1_: *P*=0.33）。Suzuki等^[52]^通过定量CT比较了两组术前及术后6个月肺容积及质量改变，认为叶切术后同侧非手术肺叶及对侧肺叶的功能代偿较肺段切除更明显，由此导致了两者术后肺功能相差无几。

病灶部位、切除肺段数量影响了段切术后肺功能保留程度。Yoshimoto等^[53]^利用SPECT/CT测量病灶同侧非手术肺叶的术后FEV_1_变化，发现下叶术后同侧非手术肺叶的FEV_1_值改变不显著。同时他们发现右上叶段切术后右中叶及右下叶的FEV_1_较术前上升，而右上叶叶切术后则呈相反的结果。随后Yoshimoto等^[53]^进一步研究了右上叶叶切或段切术后中叶FEV_1_的变化，SPECT/CT测量结果显示右上叶切除后中叶FEV_1_较术前显著降低（*P*=0.009），而右上叶段切术后中叶的FEV_1_改变较少（*P*=0.17）。该研究暗示右上叶叶切与段切术后肺功能保留的差异，不仅是因为段切切除更少的肺组织，术后中叶肺功能的改变也影响了两种术式术后肺功能保留^[54]^。Nomori等^[55]^随访117例段切患者术后FEV_1_变化，发现左上叶固有段切除与左上叶切除不存在显著差异（*P*=0.68）。对此结果他们认为是因为固有段包含的亚段数较其他肺段多，同时固有段切除可能会造成舌段的肺功能损失。有研究^[56]^指出切除的肺段越少，术后肺功能损失越少。Macke等^[57]^分析了159例患者术后6个月-36个月肺功能变化，发现FEV_1_、DLCO在切除3个-5个肺段后肺功能损失较切除1个-2个肺段多，并存在显著差异（FEV_1_: *P*=0.003; DLCO: *P*=0.015），因此他们认为较少段数的肺段切除在术后远期仍存在功能性优势。

综上所述，肺段切除在术后短期肺功能保留上较肺叶切除可能更具备优势，但需考虑到肺实质的切除程度及肿瘤位置的影响。此外，段切术后长期肺功能保留优势仍需进一步考证。

## 5 总结

随着LDCT应用于肺癌的早期筛查，早期肺癌的检出率得以提升，并可通过手术达到根治^[58]^。虽然肺叶切除仍是治疗早期NSCLC的标准术式，但肺段切除有着广阔的前景。以往的随机对照研究即LCSG^[4]^研究结果并不支持意向性段切作为首选的治疗术式，但考虑到该研究的限制性，新的随机对照研究也正在开展，如2007年启动的CALGB140503及2009年启动的JCOG0802。近期这两项研究已证实段切及叶切在围手术期死亡率及并发症发生率上相近^[43, 44]^。若CALGB140503及JCOG0802后续结果能证实对于能够耐受肺叶切除的早期肺癌患者，接受段切相比叶切在治疗早期肺癌的肿瘤学预后并不存在劣势，且在术后肺功能保留上更具备优势，则意向性肺段切除转变为治疗早期NSCLC的首选术式将具有强有力的证据。
